# The functionality of health facility governing committees and their associated factors in selected primary health facilities implementing direct health facility financing in Tanzania: A mixed‐method study

**DOI:** 10.1002/hsr2.611

**Published:** 2022-04-26

**Authors:** Anosisye Mwandulusya Kesale, Christopher Paul Mahonge, Mikidadi Muhanga

**Affiliations:** ^1^ Department of Local Government Management, School of Public Administration and Management Mzumbe University Morogoro Tanzania; ^2^ Department of Policy Planning and Management, College of Social Sciences and Humanities Sokoine University of Agriculture Morogoro Tanzania; ^3^ Department of the Development Studies Sokoine University of Agriculture Morogoro Tanzania

**Keywords:** community participation, fiscal decentralization, functionality, health facility governing committees, Lower‐Middle Income Countries, primary healthcare facilities

## Abstract

**Background:**

In Lower and Middle‐Income Countries (LMICs), decentralization has dominated the agenda for reforming the organization of service delivery (LMICs). The fiscal decentralization challenge is a hard one for decentralization. As they strive to make decisions and use health facility funding, primary healthcare facilities encounter the obstacles of fiscal decentralization. LMICs are currently implementing fiscal decentralization reforms to empower health facilities and their Health Facility Governing Committees (HFGCs) to improve service delivery. Given the scarcity of systematic evidence on the impact of fiscal decentralization, this study examined the functionality of HFGCs and their associated factors in primary healthcare facilities in Tanzania that were implementing fiscal decentralization through Direct Health Facility Financing (DHFF).

**Methods:**

To collect both qualitative and quantitative data, a cross‐sectional approach was used. The research was carried out in 32 primary healthcare facilities in Tanzania that were implementing the DHFF. A multistage sample approach was utilized to pick 280 respondents, using both probability and nonprobability sampling procedures. A structured questionnaire, in‐depth interviews, and focus group discussions were used to gather data. The functionality of HFGCs was determined using descriptive analysis, and associated factors for the functioning of HFGCs were determined using binary logistic regression. Thematic analysis was used to do qualitative research.

**Result:**

HFGC functionality under DHFF has been found to be good by 78.57%. Specifically, HFGCs have been found to have good functionality in mobilizing communities to join Community Health Funds 87.14%, participating in the procurement process 85%, discussing community health challenges 81.43% and planning and budgeting 80%. The functionality of HFGCs has been found to be associated with the planning and budgeting aspects *p* value of 0.0011, procurement aspects *p* value 0.0331, availability of information reports *p* value 0.0007 and Contesting for HFGC position *p* value 0.0187.

**Conclusion:**

The study found that fiscal decentralization via DHFF increases the functionality of HFGCs significantly. As a result, the report proposes that more effort be placed into making financial resources available to health facilities.

## 1 BACKGROUND INFORMATION

In all countries, community participation in primary healthcare (PHC) is essential for achieving excellent health and people's well‐being.^1^, ^2^, ^3^ As a result, community participation in the design, execution, and monitoring of health service delivery at PHC institutions is essential for achieving excellent health, among other things (PHC).^4^ Decentralization initiatives in Lower and Middle‐Income Countries (LMICs) allowed communities to participate in governing and administering PHC delivery. Community governance structures known as Health Facility Governing Committees (HFGCs) were created to govern Decentralization initiatives in LMICs allowing communities to participate in governing and administering PHC delivery. Community governance institutions called HFGCs were created to represent communities in the governance and management of PHC facilities.^5^, ^6^, ^7^ The newly formed HFGCs are assigned specific responsibilities and powers in the administration of primary healthcare facilities.^8^, ^9^ Following that, LMICs have continued to pursue reforms such as fiscal decentralization to empower and strengthen community engagement, or the use of HFGCs to improve health service delivery at PHC institutions.^10^ It is considered that the more empowered and autonomous HFGCs are, the more likely they are to carry out their delegated obligations, hence improving the health system's responsiveness to community needs and preferences.^6^, ^11^ Therefore, HFGC's functionality in this context entails the ability of the HFGCs to accomplish their assigned functions or duties and responsibilities.

In theory, decentralizing governance and control of health service delivery to user committees such as HFGCs improve service delivery and establish a link between healthcare professionals and communities.^8^, ^12^ However empirical studies suggest that achieving enhanced users committee's participation in governing and managing health service delivery can be very complex.^13^ Several issues related to the complexity of having effective and functional user committees or HFGCs in PHC institutions have been identified in the literature. Country context and nature of decentralization undertaken by each county are some of the cited reasons for ineffective HFGCs in PHC.^13^, ^14^ For instance, Abimbola et al.^15^ in Nigeria HFGCs were found to be underperforming in their roles because some members were unaware of their responsibilities and had the insufficient financial capacity and ability to manage facility resources. Ved et al.^16^ suggest that in India community participation through village health, sanitation and nutrition committees are not functional because they are not aligned with decentralized government. To unlock the HFGCs functionality gaps, the literature suggests the implementation of full decentralization (fiscal, political, and administrative) at PHC facilities.^17^, ^18^, ^19^ This stems from the fact that fiscal and political decentralization provides an atmosphere in which HFGCs can use their powers and fulfill their mandates. This is reinforced by the empowerment framework, which says that an agency and opportunity structure influences an individual's or group's ability to make effective decisions. The ability of an individual or group, such as HFGC, to make a meaningful decision that is influenced by their age, material ownership, abilities, experience, and educational level is referred to as agency. Opportunity structure refers to the formal or informal setting in which individuals or groups function, such as fiscal decentralization, which is determined by norms, the availability of funding, availability rules, and regulations.^20^, ^21^ Currently, some LMICs are implementing fiscal decentralization through various arrangements in primary health facilities among other things to empower and improve HFGCs' functionality.

In Tanzania, HFGCs were established in 1999 as part of Health Sector Reforms to increase community involvement in the administration and management of PHC facilities.^22^ These HFGCs are made up of members of the community who are either elected or appointed by their peers, civil society, and private health providers. The following functions are delegated from these HFGCs: Participate in the development of facility plans and budgets for the management of facility income, expenditures, and performance. Similarly, to gather funds for construction and maintenance management. Furthermore, discussing and addressing the community's concerns, as well as rallying the community to participate in the improved Health Community Fund.^22^, ^23^ However, before 2018, empirical evidence suggests that HFGCs performed poorly in carrying out their duties.^24^, ^25^ For instance, Maluka et al.^26^ *and* Kamuzora et al.^27^ found that implementation of decentralization in the district was offering only a tiny number of local elites, particularly medical professionals, were offered powers and were are allowed to participate in decision making, leaving community people and other stakeholders powerless. In other research from Tanzania, low funding, a lack of fiscal autonomy, late transfer of funds to the facility, and a lack of community participation in planning were identified as impediments to decentralization at PHC facilities.^24^, ^28^, ^29^, ^30^ To address these issues, Tanzania's government implemented Direct Health Facility Financing (DHFF) to increase fiscal decentralization at PHC facilities and allow more community/HFGC and service providers to participate in the governance and management of their health facilities at the facility level.

## 2 THE INTRODUCTION OF HEALTH FACILITY FINANCING IN TANZANIA

In 2017/18 by introduced the DHFF arrangement in all public primary health facilities. The DHFF was introduced to improve the performance of the PHC system.^22^ Under the DHFF arrangement, intergovernmental transfers for health and other funds such as Users' fees, funds from insurance schemes and development partners are directly deposited to the health facility bank accounts. The DHFF arrangement empowers service providers' and HFGCs' autonomy to plan, budget and manage facility finances to improve health services delivery.^22^

The main goal of the DHFF implementation in Tanzania was to address HFGC functionality issues such as restricted budgetary autonomy and powers, among other factors. However, there have been few studies undertaken in Tanzania to investigate the functional condition of HFGCs in the setting of DHFF. Studies on DHFF have focused on the prospect and challenges of its implementation as well as the impact of DHFF on financial management.^31^, ^32^, ^33^ Given the limitations of previous studies in informing about the status of HFGCs in carrying out their given powers and responsibilities in the context of the DHFF, this study was designed to evaluate the functioning of HFGCs in Tanzanian PHC facilities implementing the DHFF. The study is essential because the findings may be beneficial in establishing a link between fiscal decentralization empowerment and its impact on HFGCs' functioning. The findings can undoubtedly assist other developing nations in replicating fiscal decentralization in PHC institutions, whether through DHFF or otherwise.

**Figure 1:**
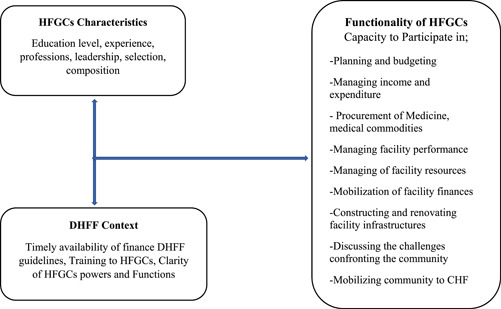
Conceptual framework

Figure 1 depicts the connection between the properties of HFGCs and the DHFF context, as well as its impact on HFGC functionality. Figure 1 implies that the functionality of HFGCs is determined by the qualities of its members, such as their education level, experience, occupation, leadership, selection, and composition. The fiscal decentralization context (DHFF) in which HFGCs operate creates a favorable setting for them to carry out their delegated tasks. The DHFF is expected to empower HFGCs by providing prompt access to funding, standards for using finance, and training for HFGCs on their roles and financial management. In addition, the DHFF framework is intended to explain HFGC's powers and mandates as they carry out their tasks and obligations. As a result of the DHFF empowerment, the HFGC's ability to carry out its responsibilities will be enhanced, and health service delivery at their facilities will improve.

## 3 METHODS AND MATERIALS

A cross‐sectional design was employed in which both qualitative and quantitative data were collected simultaneously. The study was conducted between February and May 2021 in all four regions.

### 3.1 Sample size and sampling procedure

This study used a total sample size of 280 respondents. The sample size for this investigation was determined using a four‐stage multistage cluster sampling process. Because the study encompassed geographically separated areas and face‐to‐face data collection was essential, multistage cluster sampling was used. The sample criteria were based on the Ministry of Reginal Administration and Local Government's Star Rating Assessment of all primary health facilities in Tanzania, which was completed in early 2018. Tanzania's government implemented a star rating system to assess the performance of PHC facilities, including the functionality of HFGCs. The assessed primary health facilities and their HFGC were ranked to determine the low and high‐performing health facilities and HFGCs.^34^ Star rating assessment of 2017/18 has been taken as a baseline because it is in the same year DHFF was introduced^6^, ^35^ (Table 1).

**Table 1:** Sampling process and sampling techniques

Stage	Respondent	Sampling procedure	Inclusion criteria
First Stage	Four (4) regions selected	purposive	Performance of the region in star rating assessment, Zonal representation
	Kilimanjaro, Mbeya, Ruvuma, and Songwe		
Second Stage	8 LGAs selected; Two LGAs from each region selected in stage one	purposive	Performance of the LGAs in star rating assessment, nature of the LGA (Urban and Rural),
Stage Three	32 health facilities were selected from all (8) councils; 2 health centers and 2 dispensaries from each LGA because they all implement DHFF	Stratification of health facilities into Health centers and Dispensaries Purposive selection of health centers and dispensaries	Performance of health facility (A good and poor performing health center and dispensary), Location of the facility within the LGA (Diversity)
Stage Four	280 HFGC members; 9 members from each selected health facility	Proportion sampling selection of HFGC members	members of the HFGC

Abbreviations: DHFF, Direct Health Facility Financing; HFGC, Health Facility Governing Committees.

At stage four, the representatives from HFGCs were obtained by applying the proportion sampling technique as proposed by Buddhakulsomsiry and Israel,^36^, ^37^ the formula assumed 95% confidence of level and *p* at 0.5. therefore according to the techniques the size of HFGCs members required was 288. Then the number of HFGC members from each selected health facility was determined by applying the proportional sampling technique as used by Pandev^38^ in which 9 HFGC members were supposed to be selected from each HFGC. The total simple size respondents (response) from all health facilities for this study was 280.

### 3.2 Qualitative participant recruitment and selection criteria

Qualitative recruitment of participants was done purposively. The participants who were involved in the interviews and Focus Group discussions (FGDs) were selected based on their ability to provide relevant information about the functioning of the HFGCs under DHFF and the depth of their roles in the HFGCs. Non‐HFGC participants were excluded from this study. Therefore, HFGC chairpersons were purposely selected for interviews and members of HFGC were purposively selected for FGDs. The FGDs guide was composed of 11 questions which were all about the governance of HFGC and functions of HFGCs. A total number of 14 interviews were conducted with HFGCs chairpersons and 13 FGDs were conducted with the HFGCs members composed of 6–9 participants. The number of 14 interviews and 13 FGDs were obtained after reaching the saturation point. Saturation was reached when interviewers and FGD participants kept providing similar responses therefore no new information was added through the interview.

## 4 DATA COLLECTION

### 4.1 Quantitative data collection

A closed‐ended structured questionnaire based on specific HFGC functions was used to acquire quantitative data from each selected member of HFGCs. Open Data Kit was used to construct the data gathering software (database) (ODK). After that, all of the data was entered into the ODK. A mobile data collecting quantitative approach was applied to collect data. Data was collected via mobile phones and then transmitted to a central server. Four research assistants participated in a 3‐day training session on mobile data collection skills and methodologies, which was followed by pretesting of the skills in facilities outside of the study area. The ODK platform was used to send the acquired data to the researcher. All selected facilities had GPS coordinates as part of quality control, thus all research assistants used GPS‐enabled tablets. A total of 280 out of 288 HFGCs responded to the survey.

### 4.2 Qualitative data collection

In‐depth interviews with HFGC chairpersons and FGDs involving all selected HFGC members were used to collect qualitative data. Before beginning data collecting, research assistants received training on the interview and focus group guides. Before heading to the study region, the qualitative data collection tools were tested. The interview outline included 21 questions about HFGC's functionality and governance. The 11 questions in the FGDs guide were all regarding the governance of HFGCs and their functions. HFGC chairperson's interviews involved questions like the what are main functions of HFGCs, indeed Chairpersons were required to explain how they have been accomplishing each of the functions under DHFF. HFGC members who were involved in the FGDs were required to highlight duties that they have been accomplishing in governing health facilities, their roles in implementing DHFF. FGD participants also were asked about the challenges and the factors that might help members to implement DHFF effectively.

To reduce bias in this area, we asked open‐ended questions that prevented respondents from agreeing or disagreeing. Indeed, we questioned and guided individuals to produce accurate and true information. The researcher bias was reduced by focusing on the collected data and consistently basing decisions on replies and perceptions rather than pre‐existing beliefs. The research questions were kept short and well‐organized, starting with a general topic and ending with a specific question about HFGC functionality under DHFF.

### 4.3 Quantitative analysis

The statistical program Statistical Product and Service Solution was used for the analysis (version 25). At a 5% level of significance, all statistical tests were determined. Data were analyzed using descriptive and inferential statistics, and the sample and participant characteristics were described using frequency tables and bar graphs. A binary logistic regression model was employed to determine characteristics associated with HFGC functionality because the outcome variable was dichotomized (0 = poor function, 1 = good function).^39^ The general multiple logistic regression models are given as:

$$\log\;it[\pi (x)]=\log\left(\frac{\pi (x)}{1-\pi (x)}\right)=\beta_{0}+\beta_{1}x_{1}+\text{⋅⋅⋅⋅⋅⋅}+\beta_{p}x_{p}$$

Where, π(x) is the likelihood of HFGC functionality is “good function”, $x_{i}^{\prime }s$ are set of independent variables and $\beta_{i}^{\prime }s$ are their respective parameters.^40^ The results of the model are presented in the form of a regression parameter estimate and estimated odds ratios (OR). The estimated OR, determined by taking the exponent of the regression parameter estimates, shows the increase or decrease in the likelihood of having good functionality at a given level of the independent variable as compared to those in the reference category. An estimate of OR >1 indicates that the likelihood of having good functionality for participants at a given level of the independent variable is greater than that for the reference category. Similarly, an estimate of OR <1 specifies that the chance of being having good functionality at a given level of the independent variable is less than that for the reference category.

### 4.4 Variables of the study

The dependent variable for this study was the functionality of HFGC. The Functionality of HFGCs in primary health facilities implementing DHFF was statistically analyzed based on the experience of HFGC members in accomplishing their assigned functions as indicated in the 4 points Likert Scale in which each point was in percentage. Then, the 4 points Likert scales were dichotomized for further analysis. The first two points namely “Very Low” and “Low” were coded 0 and “High” and “Very High” were coded 1. the score of functionalities was calculated by summing up all dichotomized variables. The possible minimum score was 0 and the possible maximum score was 9. The functionality score was categorized into two categories those who scored above the median (5) were regarded as good functioning while those who scored 5 or less were regarded as poor functioning. This practice is consistent with the analysis conducted in the study of health system responsiveness conducted in Tanzania.^23^ The independent variables for this study included nine (9) items (functions) which determined the functionality of HFGCs as indicated in Table 3.

### 4.5 Qualitative analysis

A total of 14 in‐depth interviews and focus groups were recorded, verbatim transcribed, and anonymised for analysis. The theme framework was employed as the theoretical framework to assess the data of HFGCs after data collection based on topic areas. The material was classified independently by four researcher assistants, and the researcher then analyzed the coded content, subcategories, and categories to determine critical conclusions. As a result, the statement referring to HFGC members' participation in various HFGC functions was studied to determine the functionality of HFGCs and to determine if the empowerment framework's argument was applicable or not.

### 4.6 Reliability

Many aspects of interest in the social sciences and other professions, such as anxiety or job satisfaction, are difficult to quantify. We ask a number of questions and integrate the answers into a single numerical value in such circumstances. When things are utilized to make a scale, however, they must be internally consistent. Cronbach's alpha was used in this study to assess our instrument's internal consistency and reliability. It assesses how well a set of variables or items accurately reflects a single, one‐dimensional latent feature of people. Cronbach's alpha values vary from 0 to 1, with values greater than 0.7 indicating adequate internal reliability. Table 3 presents Cronbach's alpha values and the number of items joined for each factor. The Cronbach's alpha value for the 9 specific functions of HFGCs is 0.922. This indicates that there is a high level of internal consistency for our scale.

The consistency of the study decision trail was used to establish qualitative dependability. The process began with the selection of assistance researchers who were well‐versed in the research issue, i.e., they had a background in community health and governance at the very least. The researchers were trained and orientated on qualitative data gathering for 3 days, as well as the data collecting method (interview and FGD tool). Data collectors did, in fact, take part in pilot research to get a sense of what's going on in the field. The environment for interviews and focus groups was setup ahead of time. To ensure privacy and uniformity, a separate room was set aside for interviews and focus groups that were far enough away from being reached or heard by healthcare practitioners. All this was done to ensure participants' freedom. apart from obtaining written consent, we sought oral consent before beginning these interviews and tried to record the oral agreement on tape. we also wanted to lighten up the tone of the interview and make it more relaxed and conversational. Through explanation and self‐determination (participants could withdraw from the study at any moment), we adhered to the ethical norms of the research procedure. The same research assistants that collected the data and had expertize with the atmosphere, participants, and reactions to the data collection process analyzed the data verbatim and transcribed the audio recordings. Some of the participants were interviewed after the data was analyzed to see if what was written matched their opinions.

### 4.7 Ethical approval

This research was carried out in conformity with the principles of the Helsinki Declaration. All procedures were followed in compliance with the applicable norms and legislation. The Sokoine University of Agriculture provided the IRB with the number SUA/ADM/R. 1/8/668. The permit was then filed to the President's Office Regional Administration and Local Government (PO‐RALG) to be granted permission to conduct research on local government authorities. PO‐RALG issued a permit with the registration number AB.307/323/01 to allow the study to conduct research in the chosen areas. All human participants in this study gave their informed consent by signing consent forms before being included in the study.

## 5 FINDINGS AND DISCUSSION

### 5.1 Sociodemographic characteristics

Table 2 shows the sociodemographic characteristics of the study's participants. The age of the members of HFGC was measured in years, the sex of the members was classified as male or female, and the educational level of the members was classified as the primary school in this study. A secondary school diploma, a certificate, a diploma, an advanced diploma, and a university degree are all options.

**Table 2:** Sociodemographic characteristics of HFGC members

		*N* = 280
Variable	Frequency	Percent
Age		
<30	32	11.43
31–45	100	35.71
46–60	107	38.21
61+	41	14.64
Sex		
Male	139	49.64
Female	141	50.36
Education level		
Primary	150	53.57
Secondary	64	22.86
Certificate	24	8.57
Diploma	30	10.71
Advanced diploma	5	1.79
University degree	7	2.50

Abbreviation: HFGC, Health Facility Governing Committee.

Table 3 shows the characteristics of the study's participants. The study's participants were divided into four regions: Mbeya, Kilimanjaro, Songwe, and Ruvuma; health facilities were divided into health centers and dispensaries; and member positions were divided into chairperson, secretary, and normal member.

**Table 3:** Number of participants as per region, type of facility, and position

		*N* = 280
Variable	Frequency	Percent
Region		
Kilimanjaro	93	33.21
Mbeya	64	22.86
Songwe	54	19.29
Ruvuma	69	24.64
Type of health facility		
Dispensary	161	57.50
Health center	119	42.50
Position		
Chairperson	43	15.36
Secretary or facility in charge	34	12.14
Member of the HFGC	203	72.50

Abbreviation: HFGC, Health Facility Governing Committee.

### 5.2 The functionality of HFGC under DHFF context

Table 4 shows the members' experiences with the nine primary functions that have been assigned to HFGCs in Tanzania. Under the DHFF context in Tanzania, HFGC members were meant to report the amount to which their HFGC had been functioning in each function in their primary health facility.

**Table 4:** HFGC functioning in various areas under decision making under DHFF, *n* = 280

Variable (specific function of HFGCs)	Poor functionality (%)	Good functionality *n* (%)	Mean (*SD*)
Participate in Preparing facility plan and Budget according to community needs	56 (4)	224 (80)	3.91 (0.92)
Managing facility income and expenditure	63 (22.49)	217 (77.5)	3.88 (1.03)
Participate in managing the procurement of healthequipment, drugs and services	42 (15)	238 (85)	4.00 (0.88)
Participate in managing facility performance	80 (28.57)	200 (71.43)	3.73 (1.05)
Management of facility resources	63 (22.49)	217 (77.5)	3.90 (0.95)
Mobilization of facility finances from different sources	118 (32.13)	162 (57.86)	3.49 (1.05)
Participate in managing constructing facility infrastructures	70 (25)	210 (75)	3.79 (1.05)
Discussing the challenges confronting the community	52 (18.57)	228 (81.43)	3.96 (0.87)
Mobilizing community to join improved Health Community Fund	36 (12.86)	244 (87.14)	4.23 (0.87)
**Overall HFGCs functioning**	**60 (21.43)**	**220 (78.57)**	**3.86 (0.79)**

Abbreviation: HFGC, Health Facility Governing Committee.

### 5.3 Factors associated with the functionality of HFGCs under DHFF context

As presented in the methodological section binary logistic analysis was used to assess factors associated with the functionality of HFGCs as presented in the methodological section. The result shows that In unadjusted analysis, the functionality of HFGCs was significantly associated with the region (*p* = 0.0456), Age of respondents (*p* = 0.0272), Education level (*p* = 0.0135), Governance (*p* = 0.0086), Health Planning aspects (*p* < 0.0001), Financial management aspects (*p* < 0.0001), Procurement Aspects (*p* < 0.0001), Informational reports (*p* < 0.0001), Measures taken by HFGC (*p* = 0.0287), Quality (*p* < 0.0001) and Important (*p* = 0.0032). After adjustment of variables, it was reviled that the functionality of HFGCs was significantly associated with Contesting position, Health Planning aspects, and Procurement Aspects and Informational reports (Table 4). With respect to Contesting position, the result showed that those HFGCs members who had contesting positions were significantly more likely to have high functionality at their health facilities as compared to those who had no contesting position (AOR = 6.413, *p* = 0.0187). With regard to Health Planning aspects, it was noted that those respondents who had good planning were significantly more likely to have good functionality as compared to those who had poor planning aspects (AOR = 10.325, *p* = 0.0011). As compared to those respondents who reported to have poor procurement aspect, those respondents who reported to have good procurement aspect were significantly more likely to have high functionality (AOR = 4.986, *p* = 0.0331). With respect to Informational reports, those HFGC members who reported to have good information reports were significantly more likely to have high functionality as compared to their counterparts (AOR = 10.387, *p* = 0.0007, see Table 5).

**Table 5:** Factors associated with the functionality of HFGCs

Variable	Unadjusted logistic regression		Adjusted logistic regression	
	OR [95% CI]	*p* value	AOR [95% CI]	*p* value
Region				
Kilimanjaro	5.137 [1.033, 25.551]	0.0456	1.950 [0.303, 12.531]	0.4817
Mbeya	0.136 [0.054, 0.343]	<.0001	8.580 [0.982, 74.960]	0.0519
Songwe	0.113 [0.044, 0.291]	<.0001	6.416 [0.854, 48.195]	0.0708
Ruvuma	Reference		Reference	
Age				
<30	Reference		Reference	
31–45	0.966 [0.410, 2.277]	0.9368	1.017 [0.233, 4.431]	0.9823
46–60	2.105 [0.859, 5.163]	0.1038	2.115 [0.421, 10.623]	0.3629
61+	4.203 [1.176, 15.025]	0.0272	1.536 [0.213, 11.061]	0.6699
How selected				
Elected	Reference		Reference	
Appointed	0.639 [0.351, 1.165]	0.1441	2.987 [0.637, 14.004]	0.1651
Contesting position				
No	Reference		Reference	
Yes	1.775 [0.989, 3.187]	0.0546	6.413 [0.749, 30.191]	0.0187
Education level				
Primary	Reference		Reference	
Secondary	1.799 [0.876, 3.693]	0.1097	1.683 [0.506, 5.592]	0.3957
Certificate	1.577 [0.554, 4.489]	0.3931	4.080 [0.747, 22.276]	0.1045
Diploma or above	3.942 [1.327, 11.706]	0.0135	6.145 [0.749, 50.430]	0.0909
Governance				
Poor	Reference		Reference	
Good	3.372 [1.362, 8.349]	0.0086	0.621 [0.100, 3.870]	0.6100
Health Planning aspects				
Not good	Reference		Reference	
Good	30.794 [14.812, 64.020]	<.0001	10.325 [2.540, 41.972]	0.0011
Financial management aspects				
Poor	Reference		Reference	
Good	17.745 [8.959, 35.148]	<.0001	1.056 [0.264, 4.223]	0.9386
Procurement Aspects				
Poor	Reference		Reference	
Good	23.364 [11.497, 47.481]	<.0001	4.986 [1.138, 21.858]	0.0331
Informational reports				
Poor	Reference		Reference	
Good	36.127 [14.675, 88.936]	<.0001	10.387 [2.671, 40.391]	0.0007
Measures were taken by HFGC				
Poor	Reference		Reference	
Good	3.882 [1.152, 13.086]	0.0287	0.463 [0.097, 2.203]	0.3335
Quality				
Poor	Reference		Reference	
Good	12.812 [5.712, 28.739]	<.0001	1.922 [0.592, 6.241]	0.2769
Important				
Poor	Reference		Reference	
Good	4.162 [1.612, 10.744]	0.0032	0.964 [0.155, 6.000]	0.9683

Abbreviations: CI, confidence interval; HFGC, Health Facility Governing Committee; OR, odds ratio.

### 5.4 The autonomy and powers of HFGCs

Participants agreed that the number of HFGCs and their fiscal powers have increased as a result of the DHFF implementation. The DHFF setup, according to the participants, has given HFGC more room to participate in planning and budgeting as well as obtaining financial resources. All these have eased the process of allocating and managing the use of allocated resources. One of the HFGC chairpersons, for example, had the following response:

*“Under DHFF arrangement member of HFGCs, we are comfortable with exerting of power in different dimensions…. It is very easy now to do what HFGC is required to do because we have all powers now” (HFGC Chairperson, Chunya DC‐ 13, February 2021)*

### 5.5 Mobilization of community to join community health insurance

HFGCs have been heavily involved in organizing communities to join CHF under the DHFF structure, according to participants in the depth interviews. Village gatherings, religious organizations, and burial rites were listed as examples of varied techniques used to organize community members to join CHF. In‐depth discussions with the HFGCs chairwoman confirmed this as well.

*“As we are speaking, CHF education is being provided to the community members, we members were divided into different groups and approached the churches found in our ward for sensitizing the community to join CHF. We have been also sensitizing communities through visiting their hamlets” (HFGC Chairperson – Madaba District Council, February 2021)*

Participants also discussed the difficulties that many communities face in recruiting community members to join CHF. Despite their commitment to this function, FDG comments indicated that the number of community members joining the upgraded community health fund is not promising in comparison to the efforts made.

*“The challenge we encounter now is the number of community members joining the CHF is very low compared to the efforts we have put in sensitizing the community about the importance of being a member of CHF*.

### 5.6 Participation in planning and budgeting process

In the implementation of the DHFF, it was discovered that HFGC engagement in planning and budgeting is high. Participants believed that under the DHFF, they no longer had to wait for council‐level planning to be completed. They revealed that they have been actively participating in the planning process through HFGC meetings, with certain members also participating through the planning committee. Participants in the focus groups described their involvement in many functions, including financial roles. The following was said by one of the FGD members.

*“We are currently able to control and monitor funds used in our facilities because we participate in deciding the use of facility funds… as HFGC chairperson, I make sure whatever we endorse to be used should also appear in the health facility plan and should be budgeted too” (HFGC Member‐ Madaba District Council‐14 February 2021)*

### 5.7 Procurement of medicine and medical commodities

Participants expressed their satisfaction with their ability to participate in the procurement of medicines and other services and items under the DHFF. They defined their involvement in the process as identifying drugs that needed to be purchased and approving the usage of monies to purchase medicines and other goods. They further stated that they had engaged in obtaining procured products and services. FGDs on the procurement process revealed the same thing.

*……when the health facility in‐charge wants to buy anything she informs us as committees, therefore we revisit our health plan and budget to see if such an item was planned to be procured…*.

Another one added

*……. The problem comes when we receive medical commodities sometimes we get stuck on the standard and quality of the materials that are to be received because we don't know how to go through them……*

### 5.8 Financial management

Participants felt that they had actively participated in the management of health institution finances under the DHFF. They highlighted the HFGC meeting as a decision‐making place where they have been discussing and making financial management decisions. They did, however, mention certain areas where they are struggling, such as raising funds from sources other than the government, health insurance, and out‐of‐pocket/user costs.

*……. In our facility, we haven't identified or solicited any other sources of finance than user fees, improved community health funds and National Health Insurance Funds… we didn't know if we were responsible for going out of what we have… (HFGC Member 2 Tunduma Town Council, March 2021)*

### 5.9 Communication between HFGCs, health workers, and community

Participants expressed favorable feelings about their relationships with health workers and communities in in‐depth interviews and focus groups. They agreed that they spoke with health workers and communities on a regular basis to identify the community's concerns. They've been collaborating with health workers to address issues in a variety of ways, including developing health plans and submitting them to village governments. This is what one of the respondents had to say:

*“We communicate with communities through several ways such as attending village assembly, meeting with individuals who have experienced some challenges in accessing health services… then we work closely with health works to address those challenges”*

## 6 DISCUSSION

The goal of this study was to evaluate the functionality of HFGCs and associated characteristics in Tanzanian PHC facilities that were implementing DHFF. In general, the study revealed that HFGCs in Tanzanian PHC facilities applying DHFF functioned well. The study also discovered that contesting the position, participation in health planning and budgeting, participation in procurement process/aspects, and discussion of various informational reports tabled in HFGC meetings are all significantly associated with the functionality of HFGCs in primary health facilities implementing DHFF.

Participants revealed that before the adoption of the DHFF in primary health institutions, they only had the political power to make decisions, but no monetary capacity to enforce such decisions. Because fiscal responsibilities remained in the hands of the councils, primary health facility plans, budgets, and procurement processes were all under their authority. HFGCs lacked the authority and autonomy to sway financial decisions based on community needs. However, after the implementation of the DHFF structure, health facilities and HFGCs have better control over their operations. Because the DHFF no longer conducts planning and budgeting at the council level, the HFGCs have been given space to participate in the planning and budgeting process. Currently, the HFGCs are in charge of approving all expenditures for facility medicine and other goods and services. Indeed, health facility management is obligated to report to the HFGC on quarterly financial, operational, and facility plan implementation status. As a result, the HFGCs have the opportunity to gather all pertinent information on facility operations and discuss it to improve healthcare delivery. These findings corroborate the empowerment framework's assertion that the capacity of a group to make successful decisions is linked to the informal and formal setting in which the group/HFGC operates.^20^, ^21^

Furthermore, as found in other studies or literature^2^, ^41^, ^42^ in lower and middle‐income nations, community participation through community governing structures is a cornerstone for enhancing health service delivery at PHC institutions. If political and administrative decentralization is not implemented simultaneously with budgetary decentralization, they are unlikely to be effective in influencing community participation in governance and managing health service delivery in catchment regions.^43^, ^44^, ^45^ The outcomes of this study back up the link between the effectiveness of community health institutions like HFGCs and fiscal decentralization via DHFF arrangements. According to this study, HFGCs are given fiscal powers and autonomy to govern PHC facilities as a result of fiscal decentralization under the DHFF arrangement.

HFGCs are functioning under the DHFF because they have been provided the opportunity to engage in planning and budgeting, procurement of medicines, medical commodities, and services, as well as through various operational reports delivered to HFGC meetings by health facility management. HFGCs may engage with communities and debate and address community health concerns through HFGC meetings, thanks to the framework provided by the DHFF setup. Other community health issues are incorporated into health facility plans and budgets to be addressed. Participants agreed that HFGCs receive all quarterly operating reports as required by DHFF guidelines under the DHFF arrangement, which helps HFGCs be more informed about their health facilities. All of these were not done or executed properly before the adoption of the DHFF.

Similarly, the findings of this study are consistent with empirical evidence from studies conducted in other countries that have implemented fiscal decentralization through DHFF, such as Kenya, which revealed that after implementing this type of fiscal decentralization, HFGC performance improved.^42^, ^46^, ^47^, ^48^ Other countries, on the other hand, implemented fiscal decentralization in PHC facilities, but HFGC functionality remained weak. In Burundi's PHC facilities, for example, fiscal decentralization was introduced, but the HFGCs' functionality did not improve.^49^ This means that depending on how fiscal decentralization is implemented, the results may vary, as in Kenya and Tanzania, where the DHFF arrangement was used, and Burundi, where payment for results was used.

Furthermore, HFGCs have been discovered to have a wide range of performance in many functions devolved to them. HFGCs have been found to have reasonably good functionality in organizing the community to join community health funds, discussing and addressing community health concerns, engaging in the procurement process, planning, and budgeting, as shown in Table 3. The DHFF's environment, in which HFGCs and facility employees are mandated to collect community health concerns and address them through facility plans and budgets, may have contributed to this.^32^ Other studies conducted before the implementation of the DHFF in Tanzania indicated minimal participation of HFGCs in discussing and addressing community health concerns, low participation of HFGCs in the planning process, and low attendance at HFGC meetings.^18^, ^50^, ^51^

The study may have underlined the relevance of fiscal decentralization in empowering subnational health organizations, particularly community governance structures like HFGCs. As the empowerment framework suggests, changing the context in which actors operate, such as through the use of the DHFF structure, improves the functionality of HFGCs, hence strengthening community engagement in the management and control of health service delivery. This is influenced by the fact that the factors found to be linked to HFGCs are also DHFF requirements. To maintain transparency and seamless operation of HFGCs, DHFF mandated HFGCs to engage in planning and budgeting, procurement processes, and health facility management to deliver quarterly reports to HFGCs Meetings. These findings contradict those of other studies, which found gender, educational levels, and other factors to be important.^1^, ^6^, ^31^, ^52^, ^53^, ^54^

## 7 CONCLUSION

The functionality status of HFGCs within the fiscal empowered environment is provided in this study, which adds to the body of knowledge and policy debate on the implementation of fiscal decentralization through DHFF. The study discovered that HFGCs in PHC facilities employing DHFF have a high level of functionality. Other studies conducted before the DHFF implementation revealed that HFGCs were underutilized and that community participation in controlling and maintaining health facilities was limited. This research has helped to find factors related to HFGC functionality under fiscal decentralization that have not been identified in earlier research. In contrast to earlier studies conducted in Tanzania that used just qualitative or quantitative methods, using a mixed‐method approach allowed researchers to obtain quantitative and qualitative findings that helped to explain the extent to which HFGCs fulfill their given duties. Future research should be performed to determine the extent to which HFGCs fulfill oversight tasks in health institutions while also conducting managerial functions.

## AUTHOR CONTRIBUTIONS

*Conceptualization, data curation, formal analysis, methodology, project administration, validation, visualization*: Anosisye Mwandulusya Kesale, Christopher Paul Mahonge, and Mikidadi Muhanga. *Investigation*: Anosisye Mwandulusya Kesale and Mikidadi Muhanga. *Resources* and *Software*: Anosisye Mwandulusya Kesale. *Supervision*: Christopher Paul Mahonge. *Writing–original draft preparation*: Anosisye Mwandulusya Kesale and Mikidadi Muhanga. *Writing–review and editing*: Anosisye Mwandulusya Kesale, Christopher Paul Mahonge, and Mikidadi Muhanga. All authors have read and agreed to the final version of the manuscript.

## CONFLICTS OF INTEREST

The authors declare no conflicts of interest.

### TRANSPARENCY STATEMENT

We confirm that this manuscript is an honest, accurate, and transparent account of the study being reported; that no important aspects of the study have been omitted; and that any discrepancies from the study as planned (and, if relevant, registered) have been explained.

## Data Availability

All data generated or analysed during this study are included in this published article and Supporting Information File.

## References

[1] Gurung G , Derrett S , Hill PC , Gauld R . Nepal's Health Facility Operation and Management Committees: exploring community participation and influence in the Dang district's primary care clinics. Prim Heal Care Res Dev. 2018;19:492‐502. 10.1017/S1463423618000026 PMC6452933 29374506

[2] WHO‐Unicef . ALMA‐ATA primary health care. Int Conf Prim Heal Care. 1978;63.

[3] World Health Organization . (2018). A Vision for Primary Health Care in the 21St Century. World Heal. Organ. (2018).

[4] Kessy FL . Improving health services through community participation in health governance structures in Tanzania. J. Rural Community Dev. 2014;9(2):14‐31.

[5] McNatt Z , Thompson JW , Mengistu A , et al. Implementation of hospital governing boards: views from the field. BMC Health Serv Res. 2014;14:178. 24742180 10.1186/1472-6963-14-178 PMC4005012

[6] McCoy DC , Hall JA , Ridge M . A systematic review of the literature for evidence on health facility committees in low‐ and middle‐income countries. Health Policy Plan. 2012;27:449‐466. 22155589 10.1093/heapol/czr077

[7] Kesale AM . Decentralization by devolution; perceptions of councilors on the level of their decision making authority in local government experience from tarime town council. J. Public Adm Gov. 2016;6:1.

[8] Mabuchi S , Sesan T , Bennett SC . Pathways to high and low performance: factors differentiating primary care facilities under performance‐based financing in Nigeria. Health Policy Plan. 2018;33:41‐58. 10.1093/heapol/czx146 29077844 PMC5886213

[9] Muhanga MI , Malungo JR . Health literacy and some socio‐demographic aspects under one health approach in Eastern Tanzania. Connections and Realities. 2018;1:89‐100.

[10] Kapologwe NA , Kalolo A , Kibusi SM , et al. Understanding the implementation of Direct Health Facility Financing and its effect on health system performance in Tanzania: a non‐controlled before and after mixed method study protocol. Heal Res Policy Syst. 2019;17:1‐13. 10.1186/s12961-018-0400-3 PMC6354343 30700308

[11] Cobos Muñoz D , Merino Amador P , Monzon Llamas L , Martinez Hernandez D , Santos Sancho JM . Decentralization of health systems in low and middle income countries: a systematic review. Int J Public Health. 2017;62:219‐229. 27572495 10.1007/s00038-016-0872-2

[12] Njoroge R , et al. It' s like these CHCs don' t exist, are they featured anywhere?”: Social network analysis of community health committees in a rural and urban setting in Kenya 2019:1‐19. 10.1371/journal.pone.0220836 PMC6687128 31393923

[13] Ramiro LS , Castillo FA , Tan‐Torres T , et al. Community participation in local health boards in a decentralized setting: cases from the Philippines. Health Policy Plan. 2001;16:61‐69. 11772991 10.1093/heapol/16.suppl_2.61

[14] Bjorkman M , Svensson J . Power to the people: evidence from a randomized field experiment on community‐based monitoring in Uganda Author (s): Martina Björkman and Jakob Svensson Published by: Oxford University Press Stable. Q J Econ. 2009;124:735‐769. URL http://www.jstor.org/stable/40506242

[15] Abimbola S , Molemodile SK , Okonkwo OA , Negin J , Jan S , Martiniuk AL . ‘The government cannot do it all alone’: realist analysis of the minutes of community health committee meetings in Nigeria. Health Policy Plan. 2016;31:332‐345. 26210167 10.1093/heapol/czv066 PMC4779146

[16] Ved R , Sheikh K , George AS , Vr R . Village Health Sanitation and Nutrition Committees: reflections on strengthening community health governance at scale in India. BMJ Glob Health. 2018;3:1‐4. 10.1136/bmjgh-2017-000681 PMC6195149 30364368

[17] Sakyi EK , Awoonor‐Williams JK , Adzei FA . Barriers to implementing health sector administrative decentralisation in Ghana: a study of the Nkwanta district health management team. J Heal Organ Manag. 2011;25:400‐419. 10.1108/14777261111155038 22039660

[18] Shayo EH , Norheim OF , Mboera LEG , et al. Challenges to fair decision‐making processes in the context of health care services: a qualitative assessment from Tanzania. Int J Equity Health. 2012;11:1‐12. 22676204 10.1186/1475-9276-11-30 PMC3476442

[19] Meyer E , Thomas L , Smith S , Scheepers C . South African health decentralisation: requiring contextually intelligent leaders. Emerald Emerg Mark Case Stud. 2017;7:1‐23.

[20] Raich U. Fiscal Determinants of Empowerment. World Bank; 2005. Policy Research Working Paper 3705. World Bank. 10.1596/1813-9450-3705

[21] Alsop R , Heinsohn N . Measuring empowerment in practice: structuring analysis and framing indicators. World Bank Policy Res Work Pap. 2005;3510:123. 10.1037/e596892012-001

[22] Kapologwe NA , Kalolo A , Kibusi SM , et al. Understanding the implementation of Direct Health Facility Financing and its effect on health system performance in Tanzania: a non‐controlled before and after mixed method study protocol. BMC Health Serv Res. 2019;17:11. 10.1186/s12961-018-0400-3 PMC6354343 30700308

[23] Kapologwe NA , Kibusi SM , Borghi J , Gwajima DO , Kalolo A . Assessing health system responsiveness in primary health care facilities in Tanzania. BMC Health Serv Res. 2020;20:1‐10. 10.1186/s12913-020-4961-9 PMC7011252 32041609

[24] WHO . Fiduciary Performance and Significant Fiduciary Risks Financial Management Risk C. Report (2015).

[25] Bakalikwira L , Bananuka J , Kigongo TK , Mukyala V . Accountability in the public health care systems: a developing economy perspective. Cogent Bus. Manag. 2017;57:1334995.

[26] Maluka SO , Hurtig AK , Sebastián MS , Shayo E , Byskov J , Kamuzora P . Decentralization and health care prioritization process in Tanzania: from national rhetoric to local reality. Int J Health Plann Manage. 2011;26:e102‐e120. 20603818 10.1002/hpm.1048

[27] Kamuzora P , Maluka S , Ndawi B , Byskov J , Hurtig AK . Promoting community participation in priority setting in district health systems: experiences from Mbarali district, Tanzania. Glob Health Action. 2013;6:22669. 24280341 10.3402/gha.v6i0.22669 PMC3841300

[28] Kilewo EG , Frumence G . Factors that hinder community participation in developing and implementing comprehensive council health plans in Manyoni District. Tanzania. 2015;9716:26461. 10.3402/gha.v8.26461 PMC4452651 26037041

[29] Frumence G , Nyamhanga T , Mwangu M , Hurtig AK . Challenges to the implementation of health sector decentralization in Tanzania: experiences from kongwa district council. Glob Health Action. 2013;6:20983. 23993021 10.3402/gha.v6i0.20983 PMC3758520

[30] Boex J , Fuller L , Malik A . Decentralized local health services in Tanzania. Urban Inst. 2015.

[31] Kesale A , Mahonge CM . The determinants of the performance of Health Facility Governing Committees (HFGC) in selected primary health facilities in Tanzania. Tanzania J. Community Dev. J. Community Dev. Prof. 2021;1.

[32] Mwakatumbula H . The implementation of Direct Health Facility Financing (DHFF): prospects and challenges. Repoa Br PB. 2021;16:1–4.

[33] Kajuni BA , Mpenzi DF . The direct health facility financing impact on financial management in Kaliua District Council, Tabora Tanzania. *World* . J Bus Manag. 2021;7:1.

[34] Yahya T , Mohamed M . Raising a mirror to quality of care in Tanzania: the five‐star assessment. Lancet Glob Heal Comm. 2018;6:1155‐1157. 10.1016/S2214-109X(18)30348-6 30196094

[35] Lodenstein E , Molenaar JM , Ingemann C , et al. We come as friends”: approaches to social accountability by health committees in Northern Malawi. BMC Health Serv Res. 2019;2:1‐14. 10.1186/s12913-019-4069-2 PMC6498677 31046748

[36] Buddhakulsomsiri J , Parthanadee P . Stratified random sampling for estimating billing accuracy in health care systems. Heal Care Manag. 2008;11:41‐54. 10.1007/s10729-007-9023-x 18390167

[37] Israel GD . Determining Sample Size 1. *Univ. Florida* . IFAS Ext. 2012:1‐5.

[38] Pandey R , Verma MR , Vulnerability C Samples Allocation In Different Strata For Impact Evaluation Of. (2008).

[39] Julie P SPSS Survial Manual A step by step guide to data analyisis using SPSS for windows (Version 12). (Open University Press, 2005).

[40] Delwiche LD , Slaughter SJ The litle SAS book. (SAS Institute Inc., SAS Campus Drive, Cary, North Carolina 27513, 2003).

[41] Lodenstein E , Mafuta E , Kpatchavi AC , et al. Social accountability in primary health care in West and Central Africa: exploring the role of health facility committees. BMC Health Serv Res. 2017;17:1‐15. 10.1186/s12913-017-2344-7 28610626 PMC5470232

[42] Waweru E , Opwora A , Toda M , et al. Are Health Facility Management Committees in Kenya ready to implement financial management tasks findings from a nationally representative survey. BMC Health Serv Res. 2013;13:1. 24107094 10.1186/1472-6963-13-404 PMC3853226

[43] The World Bank . Fiduciary Systems Assessment Tanzania – Strengthening Primary Health Care Services for Results. (2015).

[44] Smoke P . Decentralisation in Africa: goals, dimensions, myths and challenges. Public Adm Dev. 2003;23:7‐16.

[45] Fjeldstad O , Henjewele F , Mwambe G , Ngalewa E , Nygaard K . Local government finances and financial management in Tanzania with Local government finances and financial management in Tanzania. Hum. Rights. 2004;16:1‐40.

[46] Tsofa B , Molyneux S , Gilson L , Goodman C . How does decentralisation affect health sector planning and financial management? a case study of early effects of devolution in Kilifi County, Kenya Lucy Gilson. Int J Equity Health. 2017;16:1‐12. 28911325 10.1186/s12939-017-0649-0 PMC5599897

[47] McCollum R , Limato R , Otiso L , Theobald S , Taegtmeyer M . Health system governance following devolution: comparing experiences of decentralisation in Kenya and Indonesia. BMJ Glob Heal. 2018;3:1‐11. 10.1136/bmjgh-2018-000939 PMC6169670 30294460

[48] Goodman A , Opwora A , Kabare M , Molyneux S . Health facility committees and facility management—exploring the nature and depth of their roles in Coast Province, Kenya. BMC Health Serv Res. 2011;11:229. 21936958 10.1186/1472-6963-11-229 PMC3197493

[49] Falisse JB , Meessen B , Ndayishimiye J , Bossuyt M . Community participation and voice mechanisms under performance‐based financing schemes in Burundi. Trop Med Int Heal. 2012;17:674‐682. 10.1111/j.1365-3156.2012.02973.x 22487362

[50] Maluka SO , Bukagile G . Community participation in the decentralised district health systems in Tanzania: why do some health committees perform better than others? Int J Health Plann Manage. 2016;31:E86‐E104. 26044888 10.1002/hpm.2299

[51] Frumence G , Nyamhanga T , Mwangu M , Hurtig A . Participation in health planning in a decentralised health system: Experiences from facility governing committees in the Kongwa district of Tanzania 2014;9:1125‐1138. 10.1080/17441692.2014.953563 25248312

[52] Iwami M , Petchey R . A CLAS act? Community‐based organizations, health service decentralization and primary care development in Peru. J Public Health Med. 2002;24:246‐251. 12546199 10.1093/pubmed/24.4.246

[53] Roman TE , Cleary S , McIntyre D . Exploring the functioning of decision space: a review of the available health systems literature. Int J Heal Policy Manag. 2017;6:365‐376. 10.15171/ijhpm.2017.26 PMC5505106 28812832

[54] Kuwawenaruwa A , Remme M , Mtei G , et al. Bank accounts for public primary health care facilities: reflections on implementation from three districts in Tanzania. Int J Health Plann Manage. 2019;34:e860‐e874. 30461049 10.1002/hpm.2702

